# How many lobes do you see?

**DOI:** 10.1186/1749-8090-6-145

**Published:** 2011-10-26

**Authors:** Dimos Karangelis, Georgios I Tagarakis, Serapheim Chlapoutakis, Dimitrios Papadopoulos, Apostolos Roubelakis, Athanasios Hevas, Marios E Daskalopoulos, Angeliki Tsantsaridou, Stefania Lampoura, Nikolaos B Tsilimingas

**Affiliations:** 1 Department of Cardiovascular and Thoracic Surgery, University of Thessaly, Larissa, Hospital of Thessaly, Larissa, Greece; 2 Intensive Care Unit, General Hospital of Larissa, Larissa, Greece; 3 Department of Cardiothoracic Surgery, Southampton University Hospital, Southampton, UK

**Keywords:** accessory fissure, lung deformity, intraoperative image

## Abstract

Accessory fissures represent a variation of the normal lung anatomy. Incomplete development or even the absence of the major or minor fissures can lead to confusion in distinguishing adjacent lobes. This report aims to present a rare intraoperative finding of an anatomic malformation of the right lung in a 19-year old male patient with recurrent pneumothorax who underwent a surgical repair. An accessory fissure which was separating the superior segment of the lower lobe from the basal segments gave to the whole lung the unique image of a four-lobed one. A profound knowledge of the accessory fissures, even if they are incidentally discovered, is of pivotal importance for the thoracic surgeon and leads to optimal operative assessment and strategic planning.

## Introduction

Accessory fissures of the lung represent common variations of lung specimens. Several accessory fissures have been well described in time by the anatomists [1]. Accessory fissures can be described anatomically as clefts of various depth composed by two layers of visceral pleura. They may be complete or incomplete differentiating a part of lung which is termed as an accessory lobe. They are more frequently encountered in fetal and neonatal lung specimens compared to adult ones [1]. Accessory fissures often go unappreciated or misinterpreted on plain x-ray films and computed tomographic (CT) scans [2].

## Case presentation

**Figure 1:**
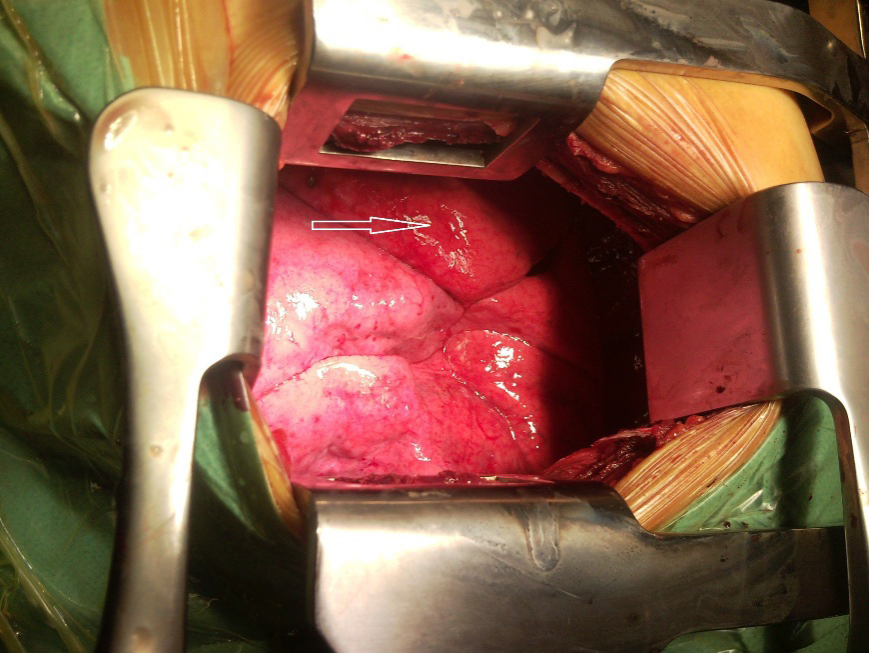
**Intraoperative image of the right lung**. The patient lies in a left lateral decubitus position with his head on the right side of the image. The superior segment of the lower lobe appears as a separate lobe (white arrow).

**Figure 2:**
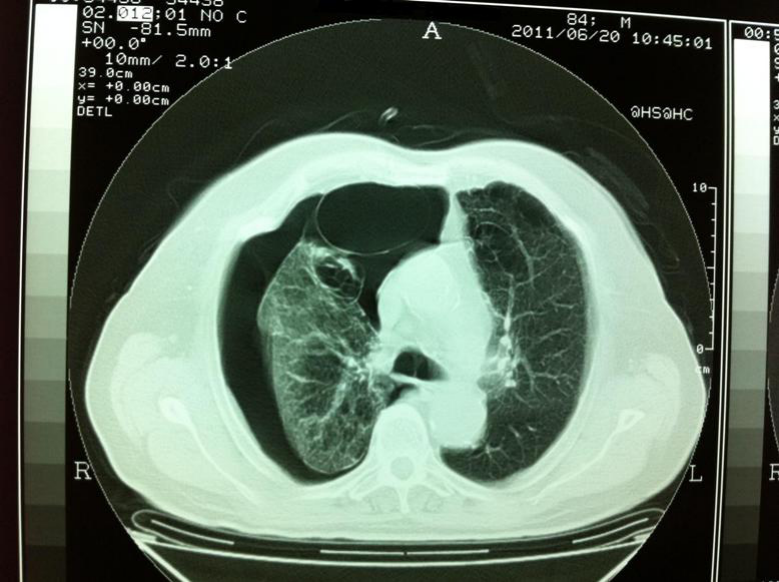
**Preoperative CT scan image of the patient (lung window) which shows the extend of bullous disease**.

A 19-year old male patient was admitted to our department suffering from recurrent spontaneous right side pneumothorax. After the insertion of the chest tube the lung reexpanded fully. Imaging of the chest revealed extended bullous disease, therefore the patient was offered the choice of surgical treatment. During the thoracotomy we discovered an interesting anatomic variation of the right lung. More specifically, the superior segment of the right lower lobe appeared separated from its native lobe through an extra fissure (Figure 1), thus giving the whole lung the image of a four lobed one. This finding was not visible in the preoperative CT (Figure 2) or plain x-ray films probably due to the patient's bullous disease. Despite the fact that thoracoscopic approach for primary spontaneous pneumothorax is considered to be the treatment of choice [3], in our case the large size of the bullae led the surgical team to avoid a VATS procedure. The patient finally underwent apical bullectomy along with partial parietal pleurectomy and chemical pleurodesis. This unique finding did not hinder the operation which was performed without any complications and the patient recovered uneventfully.

## Discussion

Supernumerary fissures are commonly encountered as variations of the lungs. On conventional CT examinations, these fissures are demonstrated infrequently due to inappropriate slice thickness, incompleteness of the fissure and the orientation of the fissure relative to the scan plane [4]. In addition, in our case the large size of the patient's bullae made even more difficult for the fissure to be recognized. The fissure we incidentally discovered was the superior accessory fissure, which separates the superior segment (S6) of the lower lobe from the basal segments. According to current literature the frequency of this aberration varies from 2% to 5% [5]. All the aforementioned studies estimate the frequency of these accessory fissures by means of high resolution CT.

In anatomic studies, reported frequency of this fissure ranges from 5 to 14% on the left, 30% on the right and 12% bilaterally [1]. When a superior accessory fissure is present, the superior segment has been called the posterior or dorsal lobe. The fissure lies at about the same level or slightly lower than the minor fissure [1].

## Conclusion

Recognition of the accessory fissures provides additional information in segmental localization of pulmonary lesions and assists in differential diagnosis of accessory fissures from normal anatomical and pathological structures [2]. Being aware of these variations before a thoracic procedure may sometimes facilitate surgical intervention. Nevertheless, in our case the fissure we encountered did not alter our surgical routine or planning and the operation was carried out without complications.

## Consent

Written informed consent was obtained from patient for publication of this report and the accompanying images. A copy of the written consent is available for review by the Editor-in-Chief of this journal.

## Conflicts of interests

The authors declare that they have no competing interests.

## Authors' contributions

DK is the main author of the manuscript and member of the surgical team. GT coauthored the paper. DP was member of the anesthesiological team. AP performed a linguistic control and helped in the revision of the manuscript. SC has been involved in drafting and revising the manuscript critically for intellectual content. AH was a member of the surgical team. AT performed linguistic control. SL has been involved in drafting the manuscript. MD checked the final version of the paper and made some crucial adjustments. NT was the primary surgeon and performed the final control. All authors read and approved the final manuscript. The manuscript is not under consideration and has not been published by another journal.
